# Cistanches alleviates sevoflurane‐induced cognitive dysfunction by regulating PPAR‐γ‐dependent antioxidant and anti‐inflammatory in rats

**DOI:** 10.1111/jcmm.14807

**Published:** 2019-12-04

**Authors:** Sheng Peng, Pengyi Li, Peirong Liu, Hongzhu Yan, Juan Wang, Weihua Lu, Chunliang Liu, Yixin Zhou

**Affiliations:** ^1^ Department of Anesthesiology Seventh People’s Hospital of Shanghai University of TCM Shanghai China; ^2^ Department of Anesthesiology Jiangsu Cancer Hospital & Jiangsu Institute of Cancer Research & The Affiliated Cancer Hospital of Nanjing Medical University & Jiangsu Red Cross Cancer Center Nanjing Jiangsu China; ^3^ Department of Pathology Seventh People’s Hospital of Shanghai University of TCM Shanghai China; ^4^ Department of Neurology Seventh People’s Hospital of Shanghai University of TCM Shanghai China

**Keywords:** cistanche, postoperative cognitive dysfunction (POCD), PPAR‐γ, sevoflurane

## Abstract

This study aimed to investigate the protective effects and underlying mechanisms of cistanche on sevoflurane‐induced aged cognitive dysfunction rat model. Aged (24 months) male SD rats were randomly assigned to four groups: control group, sevoflurane group, control + cistanche and sevoflurane + cistanche group. Subsequently, inflammatory cytokine levels were measured by ELISA, and the cognitive dysfunction of rats was evaluated by water maze test, open‐field test and the fear conditioning test. Three days following anaesthesia, the rats were killed and hippocampus was harvested for the analysis of relative biomolecules. The oxidative stress level was indicated as nitrite and MDA concentration, along with the SOD and CAT activity. Finally, PPAR‐γ antagonist was used to explore the mechanism of cistanche in vivo. The results showed that after inhaling the sevoflurane, 24‐ but not 3‐month‐old male SD rats developed obvious cognitive impairments in the behaviour test 3 days after anaesthesia. Intraperitoneal injection of cistanche at the dose of 50 mg/kg for 3 consecutive days before anaesthesia alleviated the sevoflurane‐induced elevation of neuroinflammation levels and significantly attenuated the hippocampus‐dependent memory impairments in 24‐month‐old rats. Cistanche also reduced the oxidative stress by decreasing nitrite and MDA while increasing the SOD and CAT activity. Moreover, such treatment also inhibited the activation of microglia. In addition, we demonstrated that PPAR‐γ inhibition conversely alleviated cistanche‐induced protective effect. Taken together, we demonstrated that cistanche can exert antioxidant, anti‐inflammatory, anti‐apoptosis and anti‐activation of microglia effects on the development of sevoflurane‐induced cognitive dysfunction by activating PPAR‐γ signalling.

## 1 INTRODUCTION

Postoperative cognitive dysfunction (POCD) is a devastating complication with long‐lasting consequences, and is defined as the impairment of memory, attention, concentration and information handling occurring after surgery and anaesthesia. Researches have indicated that about 10% of surgical patients and nearly 40% of elderly patients at age 65 and even older will experience POCD.^1^ Patients with POCD show strikingly higher mortality, reduced quality of life and elevated dependency on social security when compared to those without POCD.^2^, ^3^ Although numerous research efforts had been made in recent decades, the pathogenesis of POCD remains largely unknown.

It is well recognized that neuroinflammation and oxidative stress in the brain play crucial role in the initiation and progress of POCD.^4^, ^5^ Although surgery‐induced inflammation or oxidative stress can cause neuroinflammation and damage in the brain and ultimately contribute to cognitive disorder, recent reports suggest that anaesthetic agents used during the surgery are also capable of triggering cognitive impairment.^3^, ^6^, ^7^

Sevoflurane is commonly used in clinical anaesthesia for patients of all ages due to its low blood‐gas partition coefficient and low metabolic breakdown.^8^ However, several lines of evidence have shown that sevoflurane could lead to neuroinflammation and impair cognitive function. For example, Makaryus reported that sevoflurane has neurotoxic effects on the development of brain.^9^ Decreased connectivity between excitatory neurons in the prefrontal cortex has been shown to be correlated with cognitive impairments induced by sevoflurane.^10^ Homoplastically, sevoflurane facilitates cognitive decline by aggravating microglia‐regulated neuroinflammation in a rat model via down‐regulating PPAR‐γ activity in the hippocampus.^1^ Once the surgical method is determined, the degree of trauma and inflammatory response cannot be changed. Therefore, reducing cognitive impairment from the perspective of anaesthesia may become a feasible approach.

The cistanche species (‘Rou Cong Rong’ in Chinese) has been used as a tonic in China for many years. Modern pharmacological studies have demonstrated that cistanches possesses broad medicinal functions, especially on the central nervous system (CNS), including anti‐apoptosis, anti‐oxidation, anti‐ageing, anti‐fatigue, immunomodulatory anti‐inflammatory and neuroprotection.^11^, ^12^, ^13^ Deng et al found that the echinacoside, one of the major active compounds of cistanche, exhibited protective effects on TNF‐α‐induced SH‐SY5Y cell apoptosis.^14^ Phenylethanoid glycosides, another major active compounds of cistanche, which provided abundant phenolic hydroxyl, can serve as hydrogen donor to reductive radicals and thus scavenge them. There are two main flows by which the cistanches scavenge the free radicals, namely directly involving in the removal of free radicals or blocking their production and regulating the antioxidant enzymes related to the free radical metabolism in vivo, such as SOD, CAT and GPX.^15^ Cistanches administration contributes to slowing ageing phenotypes and relieving cognitive decline in flies by reducing oxidative stress.^16^ For instance, Zhang et al investigated one of the cistanches extractions and confirmed its function of extending lifespan.^17^ To date, however, no study examined the effects of cistanches on cognitive dysfunction in either animal models or humans under sevoflurane conditions.

Based on above findings, we suggested that cistanches could attenuate cognitive impairment induced by sevoflurane. To verify this hypothesis, we used 24‐month‐old SD rats to get cognitive impairment model by inhaling sevoflurane. Subsequently, we explored the pharmacological function of cistanches extract on anti‐inflammatory, anti‐oxidation and neuroprotection. And the mechanism of cistanches on cognitive impairment induced by sevoflurane was also investigated.

## 2 MATERIALS AND METHODS

### 2.1 Preparation of Cistanches Herba extract and standardization

The extraction of cistanches was according to previous works.^18^, ^19^ Briefly, Cistanches Herba extract was extracted with 95% ethanol in water and lyophilized to the powder. The extract at 10 mg/ml was applied on quantitative chemical analysis of echinacoside by reverse‐phase high‐performance liquid chromatography (HPLC). Standard compound, echinacoside, was obtained from Haoxuanbio (Xi'an, Shaanxi, China).

### 2.2 Rat model of sevoflurane‐induced cognitive dysfunction and treatment with cistanche

Male Sprague Dawley (SD) rats aged 3 and 24 months were purchased from the Beijing Vital River Laboratory Animal Technology Co., Ltd. Rats were raised in clean acrylic cages with free access to water and food under 12‐hour light/dark cycle at a constant temperature of 21°C ± 1°C and humidity (45%‐65%) for 1 week before the experiment for accommodation. The animal experimental procedures were authorized by the Animal Care Committee of Seventh People's Hospital of Shanghai University of TCM. All behavioural tests were carried out in a temperature (21°C ± 1°C)‐ and humidity (45%‐65%)‐controlled room.

In the first set of experiments in vivo, the 3‐ and 24‐month‐old male SD rats were used. Sixteen 3‐month‐old rats weighing 200‐250 g and sixteen 24‐month‐old rats weighing 240‐300 g were randomly assigned to indraft sevoflurane (Jiangsu Hengrui Medicine Co., Ltd., Lianyungang, China) or carrier gas (n = 8 for each group). Animals in the anaesthesia groups (SEVO) were subjected to 2.6% concentration of sevoflurane delivered by a humidified 30% O_2_ carrier gas for 4 h at a flow rate of 2 L/min using an anaesthetic apparatus with a multi‐gas monitor (Prisma SP Alpa, Oxon, UK). Rats in the control group were subjected to the carrier gas without sevoflurane for the same time.

In the second set of experiments, 24‐month‐old male SD rats were either administered or not with 50 mg/kg cistanche orally before being subjected to anaesthesia by inhaling of 2.6% sevoflurane for 4 hours (n = 8 for each group). The behavioural tests were conducted on day 1 and day 3 after anaesthesia. Then animals were killed immediately after behavioural assessment at day 3, and hippocampus in the brains was used for molecular analyses. In the third set of experiments, all anaesthetized rats were randomly divided into four groups (n = 8 for each group): (a) SEVO group; (b) SEVO + GW9662 (an antagonist of PPAR‐γ, 30 ng every time) group; (c) SEVO + cistanche group; and (d) SEVO + cistanche + GW9662 group. The subsequent experiments were similar to the description in the second set of experiments.

### 2.3 Behavioural testing

Morris Water Maze Test was performed as described previously with slight modification to evaluate the spatial memory and learning abilities of rats in the present study.^8^, ^20^ A tank was filled to a depth of 40 cm with water and made opaque by adding nontoxic white tempera paint. Water temperature was maintained at 22 ± 1°C to prevent the mouse from floating. A circular escape platform (11 cm radius) was placed 0.5‐1 cm below the water surface in one of four quadrants. At three time‐points, rats were tested for reference memory after training for 5 days and each training trial section consisted of four trials with an interval of 15 minutes. At the second and third time‐point, the platform was removed from the pool, then spatial and working memory measurement was conducted. The time each rat spent to look for the platform was recorded as the escape latency. After trials, rats were put in heated cages before returned to their cage.

The open‐field test was carried out at day 1 and day 3 post‐anaesthesia as described by Fricano *et al* 21 Briefly, rats were tested in a 27.5 cm × 27.5 cm square arena. The middle, 66% of the arena, was entitled as the ‘centre’. The time spent in the centre was recorded as the parameter to evaluate the anxiolytic behaviour.

The fear conditioning test was performed as described previously with slight modification to explore the spatial memory and learning abilities of rats in the present study.^22^, ^23^ The training was performed on one day after anaesthesia. Each rat was allowed to adapt to the chamber for 120 s, followed by exposure to a sound stimulus (20 s, 80 dB) and then an electric foot shock (2 s, 0.75 mA) 25 s after the sound stimulus ended. The exposures were repeated twice, with 60‐s intervals between the second and third exposures. The context test was performed on the second and third days after surgery. Each mouse was put in the same chamber used for training, followed by no stimulus and no foot shock for the same interval used to train the animal. The results were recorded, and the percentage of freezing time (not moving) was collected.

### 2.4 Tissue collection

After behavioural analysis at day 3 post‐anaesthesia, the experimental rats were killed; then, the brain tissues were removed immediately and hippocampus was dissected carefully. For the proteins, RNAs and cytokines analysis, the hippocampus was frozen in liquid nitrogen immediately and stored at −80°C. For immunohistochemical analysis, tissues were fixed with 4% paraformaldehyde for 72 hours and then transferred to 20% sucrose for another 72 hours.

### 2.5 Enzyme‐Linked Immunosorbent Assay

All rats were killed after the behaviour test at day 3. Hippocampal tissues were dissected on ice immediately and homogenized in saline by ultrasonic. Then, centrifugation was performed at 10 000 × *g* at 4°C for 15 minutes. Levels of IL‐1β, TNF‐α and IL‐6 were determined by enzyme‐linked immunosorbent assay (ELISA) kits (eBioscience, San Diego, CA, USA) according to the manufacturer's instructions. Detection limits were 8.0 pg/mL for IL‐1β, 8.0 pg/mL for TNF‐α and 4.0 pg/mL for IL‐6. Plasma IL‐1β, TNF‐α and IL‐6 concentrations at day 1 and day 3 were also measured using an ELISA.

### 2.6 Determination of nitrite/nitrate and oxidative damage to lipids and proteins

The nitrite/nitrate (NO2^−^) concentration in the collected supernatant was measured by the Griess reaction (Sigma, St. Louis, MO, USA), which showed as an indicator of the production of nitric oxide (NO). Briefly, 100 μL of Griess reagent was added to 100 μL of the supernatant. The total protein content of the supernatant was measured with a BCA protein assay reagent kit (KeyGEN, China) after incubation at room temperature for one hour, and the absorbance was measured using a spectrophotometer at 550 nm. Results are nmol of NO_2_^−^ per mg of protein.

The level of malondialdehyde (MDA) in the hippocampus was determined by the MDA assay kit (Jiancheng Bioengineering Institute, Nanjing, China) after collected via homogenate and centrifugation. The oxidative damage to lipids was determined by the thiobarbituric acid reactive substances (TBARS) assay. Briefly, the supernatant samples were precipitated with 10% trichloroacetic acid. Then thiobarbituric acid (0.67%) was added, and the absorbance was tested at 535 nm. The 1,1,3,3‐tetramethoxypropane as an external standard and data were expressed as nmol of malondialdehyde equivalents per mg of protein.

### 2.7 Antioxidant enzyme activity detection

For the measurement of superoxide dismutase (SOD) activity, 10 μL of supernatants obtained by tissue homogenizing and centrifugation (10 000 × *g*, 15 minutes) at 4°C was added to 200 μL reaction solution (50 μL tetrazolium plus 19.95 mL assay buffer, including 50 mM Tris‐HCl, 0.1 mmol/L hypoxanthine and 0.1 mmol/L diethylene triamine pentaacetic acid, pH 8.0). Then, 20 μL of xanthine oxide was added to the mixtures to initiate the reaction. After shaking and incubating for 20 minutes, absorbance changes at 450 nm were recorded using a spectrophotometer. The SOD activities were calculated using the formula supplied with the detecting kit (Cayman Chemical Company, Ann Arbor, MI, USA).

The CAT activity in hippocampal tissue supernatant was determined spectrophotometrically by calculating the hydrogen peroxide (H_2_O_2_) decomposition rate at 240 nm. Briefly, an aliquot of 100 μL of the supernatant (20 μL) was added to the substrate mixture (1000 μL) containing 0.3 ml of H_2_O_2_ in 50 mL of 0.05 mol/L PBS (pH 7.0). Absorbances were recorded at 1 minute after the initiation of the reaction. The results were calculated using external standard and expressed as U per mg of protein.

### 2.8 RNA isolation and quantitative PCR

The hippocampal tissues were homogenized to obtained total RNAs using TRIzol (Invitrogen), and then, transcript amplification was implemented using iScript cDNA Synthesis Kit (Bio‐Rad). The resulting cDNA was amplified by qPCR using iTaq Universal SYBR Green Supermix (Bio‐Rad) with gene‐specific primer primers, and the relative expression of each gene was calculated using the 2^−ΔΔCt^ method and normalized to GAPDH control. The primer sequences are presented in Table S1.

### 2.9 Western blot

The hippocampal tissues were homogenized in cell lysis buffer (50 mmol/L Tris, pH 7.5, 1 mmol/L EDTA, 150 mmol/L NaCl, 0.5% Triton X‐100, 0.5% NP‐40, 1 mmol/L phenylmethylsulphonyl fluoride, 5 mmol/L sodium vanadate, 1 mmol/L sodium fluoride, 1 μg/mL of aprotinin, pepstatin and leupeptin). Proteins were separated by SDS‐PAGE gels, and then, proteins in the gels were transferred to PVDF membranes (Millipore). The membranes were blocked with 5% milk and then incubated with specific primary antibodies, including anti‐β‐APP (ab76763, 1:1000 dilution), anti‐BDNF (ab108319, 1:1000 dilution), anti‐GFAP (ab68428, 1:1000 dilution), anti‐Iba‐1 (ab178847, 1:1000 dilution), anti‐PPAR‐γ (ab209350, 1:1000 dilution), anti‐NF‐κB (ab32360, 1:1000 dilution) and anti‐GAPDH (ab181602, 1:1000 dilution). Subsequently, TBST was used to wash the membranes and then incubated it with HRP‐conjugated secondary antibodies (Santa Cruz, CA, USA). Finally, the relevant protein was observed by ECL system (PerkinElmer) in the light of the manufacturer's explication. And ImageJ was applied for quantitative analysis of relative protein expression.

### 2.10 Fluorescence‐activated cell sorting (FACS) analysis

For FACS analysis, the hippocampal cells were collected after isolation immediately. Then, the cells were washed using ice‐cold phosphate‐buffered saline (PBS) and resuspended in PBS containing 2% FBS. For each assay, 1 × 10^5^ cells in 100 µL PBS‐FBS were stained with fluorescently conjugated CD11b (BD PharMingen, catalog #554982, 1:250) and CD45 (BD PharMingen, catalog #554878, 1:250) antibodies on ice for 2 hours at 4°C in the dark. After washing with cold PBS, cells were resuspended in 300 µL PBS‐FBS. And flow cytometric analysis was performed on a FACSAria I flow cytometer (BD Biosciences). Results were analysed using FlowJo (version 7.6.1).

### 2.11 Nissl staining

The collected hippocampus was embedded in OCT compound, and then cut into 14‐μm coronal sections using a freezing microtome (Leica). After thaw‐mounting on charged slides, the sections were washed with 0.01 mol/L PBS three times for total 15 minutes and then incubated in cresyl violet solution (0.5%, containing a few drop of glacial acetic acid) for 10‐15 minutes at room temperature. After washing with distilled water for twice, the sections were dehydrated in ethanol gradually (70%, 85%, 95% and 100%). Following dehydration, they were placed in xylene and coverslipped using a mounting medium. Finally, the Nissl staining was observed under the microscope (Leica).

### 2.12 Data analysis

Data were analysed using the GraphPad Prism 6.0 software. Data in the behaviour studies were analysed at different time‐points (day 1 or day 3) after sevoflurane anaesthesia using two‐way analysis of variance (ANOVA) with repeated measures. Other data were analysed using one‐way ANOVA before the Bonferroni test. All experiments were performed in triplicate, and the results were expressed as the mean ± SEM *P* < .05 was considered statistically different.

## 3 RESULTS

### 3.1 Cistanche prevented abnormal neuroinflammation in aged rats

To investigate the difference in cognitive function between adult and ageing rats, sevoflurane was used to induce anaesthesia on the 3‐ and 24‐month‐old rat. Then, the hippocampus‐dependent behaviour was evaluated on day 1 and day 3 (Figure S1A). As shown in Figure S1B, there was no difference in escape latency for four experimental groups on day 1. Furthermore, the time spent in the centre and the per cent of freezing were similar to rats that received vehicle and sevoflurane on day 1 (Figure S1C, D). However, the 3‐ and the 24‐ month‐old rats had significant difference in the escape latency, time spent in the centre and the percentage of freezing after the sevoflurane induction on day 3, suggesting that sevoflurane altered the cognitive function of aged animals significantly (Figure S1B‐D). Thus, the aged rats were used for the subsequent experiments.

**Figure 1:**
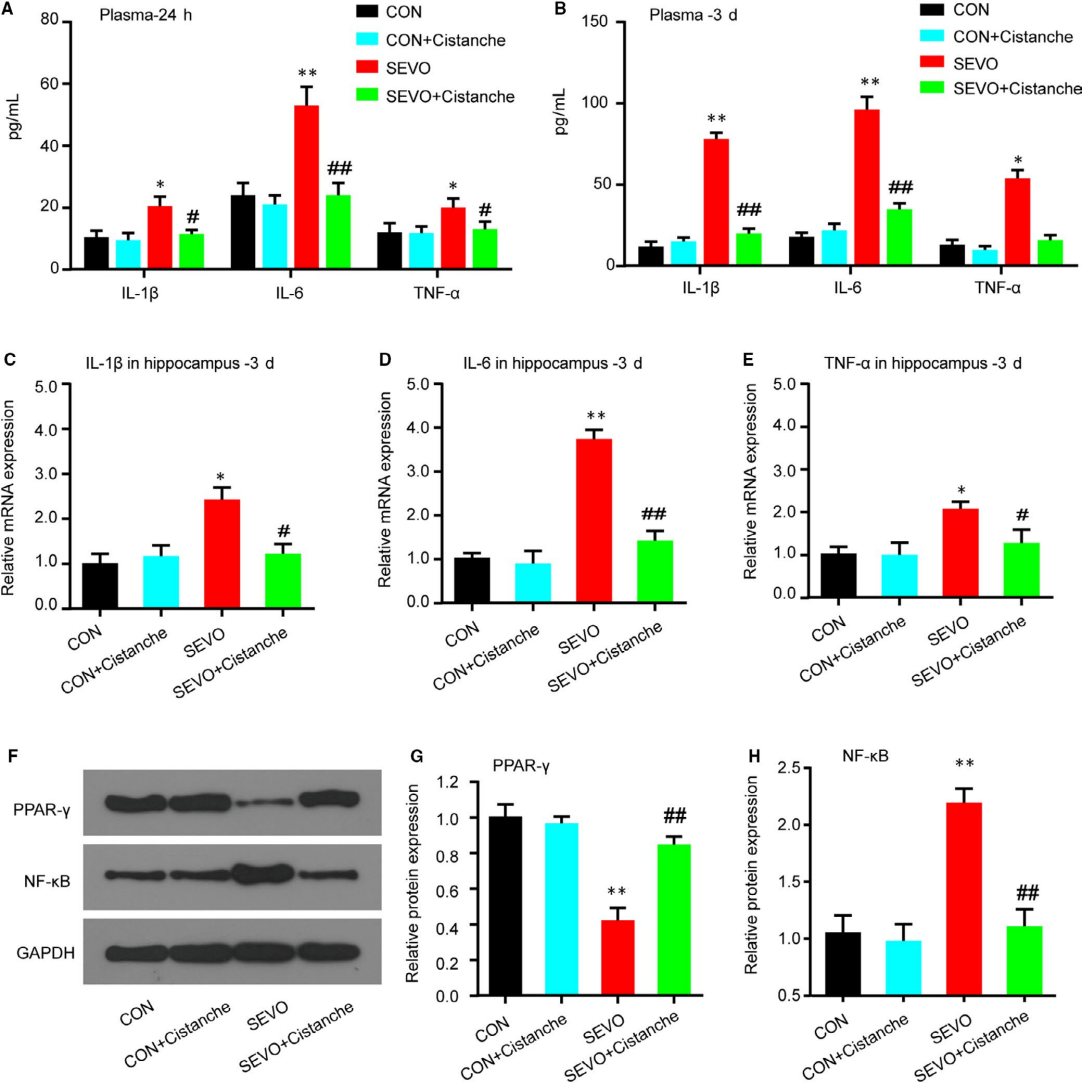
Cistanche administration attenuates abnormal neuroinflammation in sevoflurane‐induced rats. A, B, ELISA analysis for the concentration of IL‐1β, IL‐6 and TNF‐α in rat plasma samples on day 1 (A) and day 3 (B) after treatment. C‐E, Relative mRNA levels of pro‐inflammatory cytokines IL‐1β, IL‐6 and TNF‐α in the hippocampus on day 3 after treatment were evaluated by qRT‐PCR. (F) The PPAR‐γ and NF‐κB protein expression in the hippocampus was detected by Western blot. G, H, Representation of PPAR‐γ/GAPDH and NF‐κB/GAPDH at day 3 time‐points. Data are mean ± SD (n = 8 per group). *P < .05, **P < .01 vs CON group; ^#^ *P* < .05, ^##^ *P* < .01 vs SEVO group

To investigate the effects of cistanche on the neuroinflammation in aged rats undergoing sevoflurane exposure, we evaluated the pro‐inflammatory cytokines levels. The evidence for neuroinflammation was seen at 24 hours after sevoflurane anaesthesia, as demonstrated by high concentration of pro‐inflammatory cytokines IL‐1β, IL‐6 and TNF‐α in the plasmid on day 1 and day 3 (Figure 1A, 1), and the neuroinflammation was indicated by the elevated mRNAs of pro‐inflammatory cytokines in the hippocampus up to 3 day (Figure 1C‐E). For each of the pro‐inflammatory factors, cistanche treatment eliminated the differences in the sevoflurane‐induced changes in the proinflammatory mediators. Cistanche significantly attenuated the elevated pro‐inflammatory cytokines in the sevoflurane anaesthesia aged rats. However, those pro‐inflammatory cytokines in control + cistanche group showed no significant differences against control group (Figure 1A‐E). We also observed changes in PPAR‐γ and NF‐κB protein in the hippocampus within 3 days after the induction. In the group SEVO + cistanche, the expression level of PPAR‐γ was dramatically increased and that of NF‐κB was significantly reduced on day 3 post‐induction compared to that in group SEVO (Figure 1F‐H). These results indicated that cistanche could at least partially inhibit neuroinflammation after sevoflurane anaesthesia.

### 3.2 Cistanches Herba extract treatment attenuated sevoflurane‐induced oxidative stress in hippocampus of aged rats

**Figure 2:**
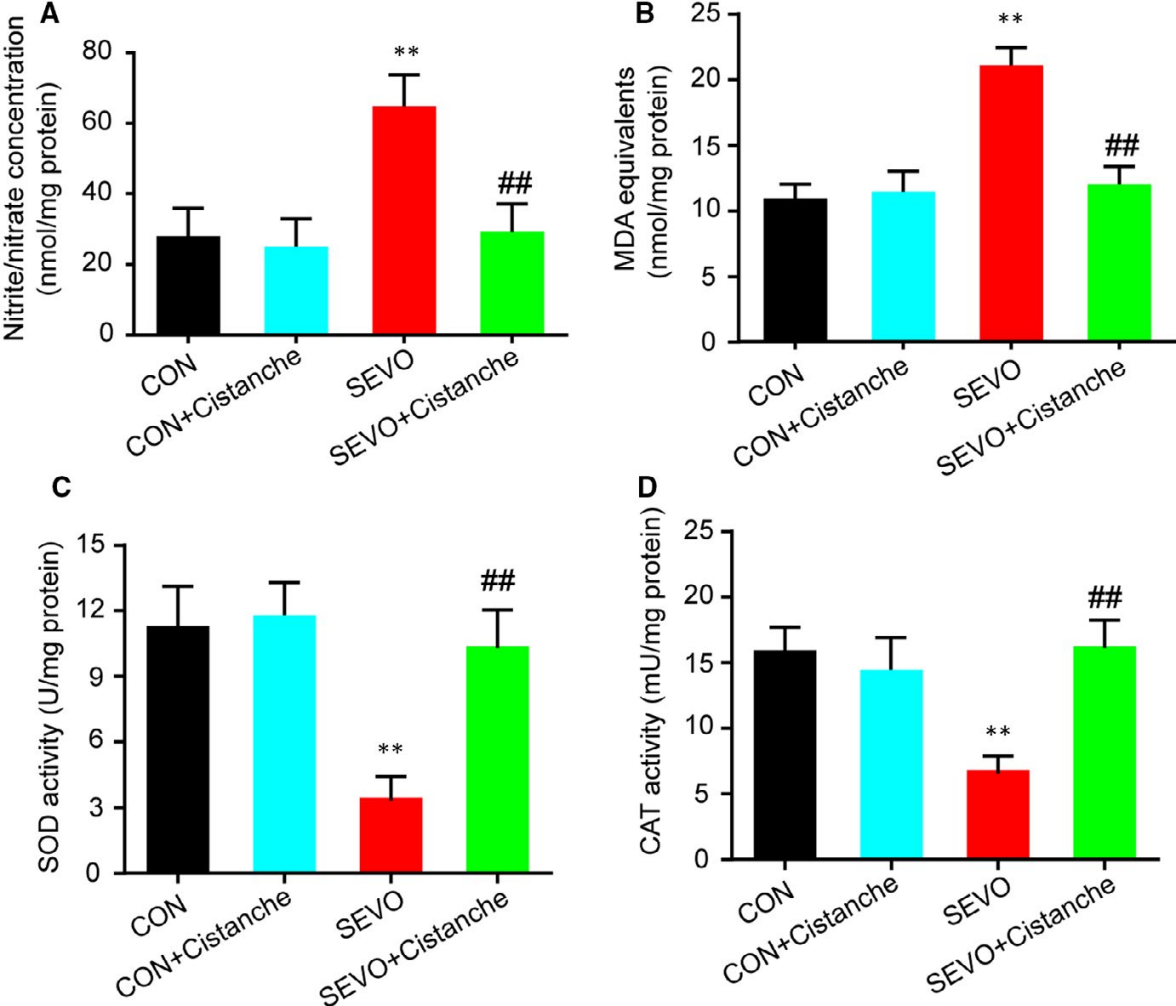
Cistanche inhibits the oxidative stress induced by the sevoflurane. A, Nitrite/nitrate concentration in hippocampus was measured by the Griess reaction. B, Lipid peroxidation in hippocampus was determined using TBARS assay. C, Superoxide dismutase activity in hippocampus was measured using a xanthine oxide method. D, Catalase activity in hippocampus was measured by calculating the hydrogen peroxide (H2O2) decomposition rate at 240 nm. Data are expressed as mean ± SD (n = 8 per group), **P < .01 compared to CON group; ^##^ *P* < .01 vs SEVO group

Next, we investigated the effects of cistanche on the oxidative stress induced by sevoflurane. As illustrated in Figure 2A, we found that the cistanche treatment prevented sevoflurane‐induced oxidative stress, as suggested by significantly decreased concentration of nitrite/nitrate in the hippocampus. We also observed that the SEVO + cistanche group presented lower levels of MDA equivalents, an index of the oxidative damage to lipids, in the hippocampus at three days after the sevoflurane inhalation, compared to the SEVO group (Figure 2B). In addition, rats subjected to sevoflurane anaesthesia (SEVO group) presented a significant decrease in SOD and CAT activity on day 3 after anaesthesia in the hippocampus. However, the hippocampal activity of those enzymes was significantly increased in SEVO + cistanche group, compared to the SEVO group (Figure 2C, D).

### 3.3 Cistanches Herba extract treatment attenuated microglia activation

**Figure 3:**
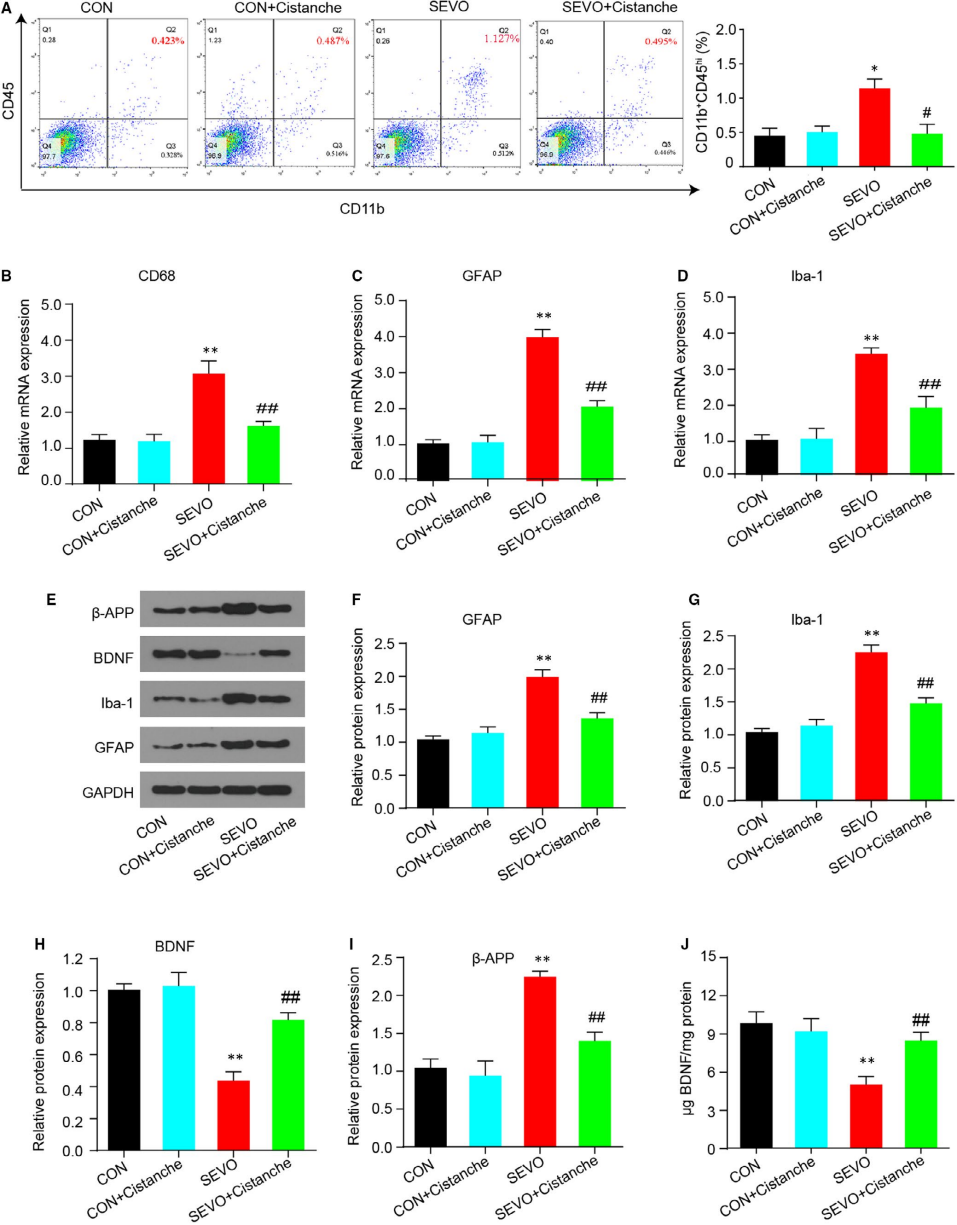
Cistanche inhibits microglia activation in the hippocampus induced by the sevoflurane. A, Microglia analysed by two‐colour flow cytometry displayed different patterns of CD11b and CD45 expression in hippocampus of rats treated with cistanche or saline. Region 2 shows the percentage of the subpopulation of CD11b^+^CD45^hi^‐activated microglia. And the percentages of activated microglia over total cells were quantified. B‐D, The glial markers CD68, GFAP and Iba‐1 were measured by qRT‐PCR. E‐I, Western blot assay for β‐APP, BDNF, Iba‐1 and GFAP protein levels on day 3 after cistanche administration. J, BDNF levels in the hippocampus of aged rats subjected to cistanche administration were examined. Data are expressed as mean ± SD (n = 8 per group), *P < .05, **P < .01 vs CON group; ^#^ *P* < .05 and ^##^ *P* < .01 vs SEVO group

Microglial cells are activated during the laboratory or anaesthesia‐induced inflammation in the hippocampus, which then contributes to the pathological progress. To investigate whether cistanche could prevent the microglia activation, we measured the population of activated microglia cells and its relative markers. Resting microglia expressed CD11b and a low expression of CD45, while activated microglia expressed CD11b and high level of CD45. As presented in Figure 3A, results from flow cytometry analysis confirmed that the percentage of CD45 was significantly lower in cistanche‐treated rats than in SEVO‐induced rats (0.495 ± 0.051% following cistanche treatment vs 1.127 ± 0.032% in the SEVO group). Subsequently, we measured the glial markers by qRT‐PCR. After anaesthesia with sevoflurane, the mRNA level of CD68, GFAP and Iba1 was increased in the hippocampus of aged rats at 3 d after induction. Notably, about twofold decrease in expression of mRNA for CD68, GFAP and Iba1 was observed in SEVO + cistanche rats compared with the SEVO group at day 3 after induction (Figure 3B‐D). Figure 3E‐G shows graded, significant down‐regulation of GFAP and Iba1 protein in the SEVO + cistanche (vs SEVO group). To further explore neuronal injury, we then detected the expression levels of BDNF and β‐amyloid precursor protein (β‐APP). We observed that sevoflurane anaesthesia resulted in a significant increased β‐APP and down‐regulated BDNF protein levels in the SEVO group. However, additional cistanche treatment suppressed β‐APP level and elevated BDNF level of rats compared to those subjected to sevoflurane exposing along (Figure 3E, H, I). Also, ELISA performed after 3 days of treatment with cistanche revealed a strong increase in BDNF concentration, confirming inhibition of nerve injury in vivo (Figure 3J). Collectively, these results suggested that cistanche treatment prevented microglial activation.

### 3.4 Cistanches Herba extract treatment attenuated sevoflurane‐induced neuronal apoptosis

**Figure 4:**
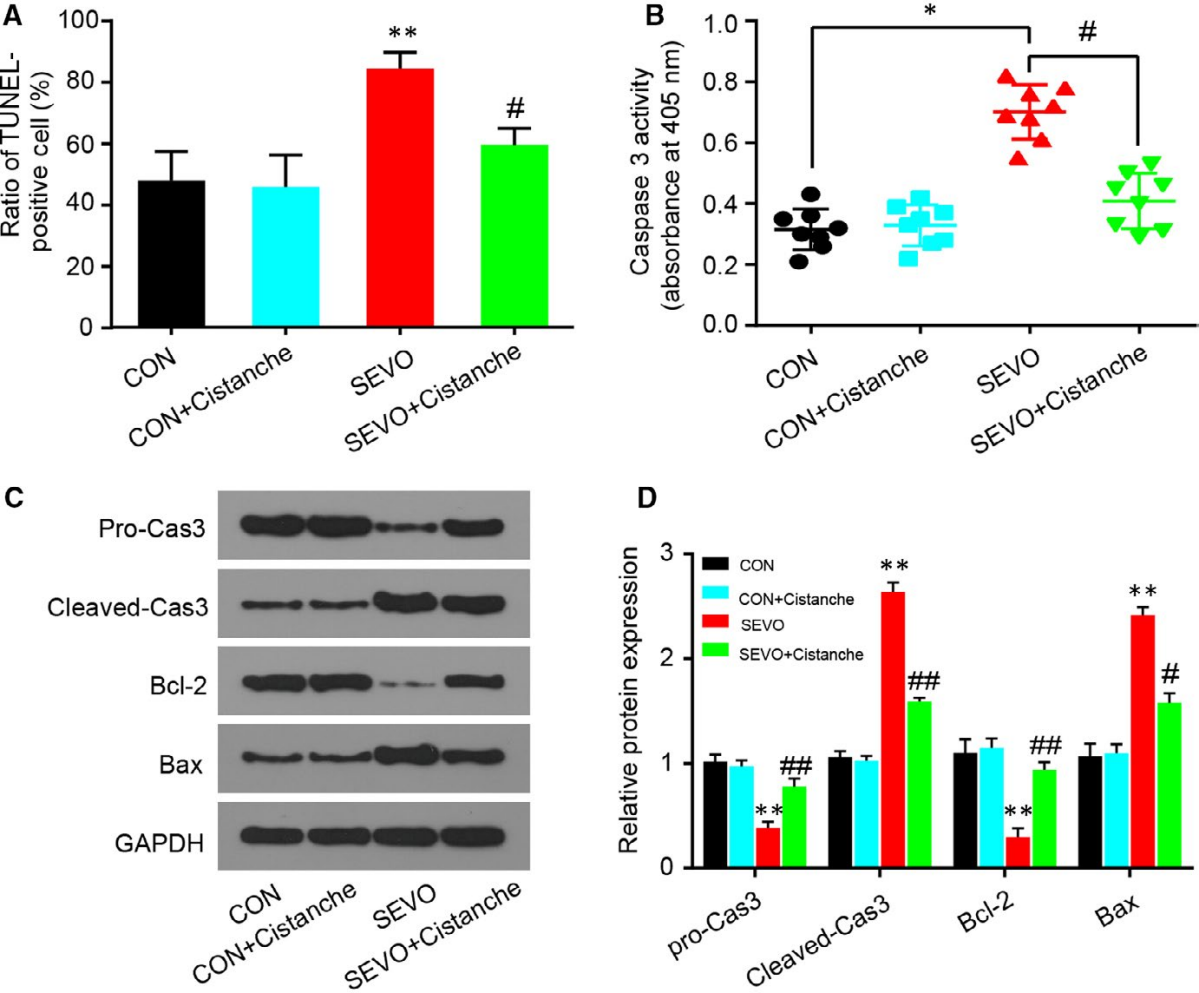
Cistanche inhibits sevoflurane‐induced neuronal apoptosis in the hippocampus. A, Ratio (%) of TUNEL‐positive cells relative to DAPI‐positive total nuclei in the hippocampus is indicated in the histogram. B, Caspase 3 enzymatic activity assay in the hippocampus of anaesthetized rats untreated or treated with cistanche for 3 days. C, The expression of pro‐caspase 3, cleaved‐caspase 3, Bcl‐2 and Bax in the hippocampus of rats was detected by Western blotting using specific antibodies. D, Expression of pro‐caspase 3, cleaved‐caspase 3, Bcl‐2 and Bax was quantified and normalized to GAPDH levels. Data are expressed as mean ± SD (n = 8 per group), *P < .05 and **P < .01 vs CON group; ^#^ *P* < .05; ^##^ *P* < .01 vs SEVO group

Neuronal apoptosis is partial driven by activated microglia and oxidative stress, and we had confirmed that cistanche could prevent microglia activation and oxidative stress in vivo. To further investigate the pharmacological effects of cistanche, we tested for neuronal apoptosis with TUNEL staining and cleaved‐caspase 3 and Bax/Bcl‐2 immunoblot. As presented in Figure 4A, sevoflurane increased cell apoptosis in the hippocampal regions. Cistanche administration reduced TUNEL^+^ cells following sevoflurane anaesthesia. We also illustrated the remarkable high levels of caspase 3 and Bax, while low level of Bcl‐2 in the hippocampus of anesthetized aged rats. Rats that received administration of cistanche before anaesthesia revealed significant reduction of caspase 3 activity (Figure 4B), cleaved‐caspase 3 and Bax protein (Figure 4C, D), which was consistent with reduced neuronal apoptosis observed in TUNEL staining.

### 3.5 Cistanches Herba extract treatment alleviated cognitive dysfunction and impairment of hippocampal tissue following sevoflurane‐induced anaesthesia

**Figure 5:**
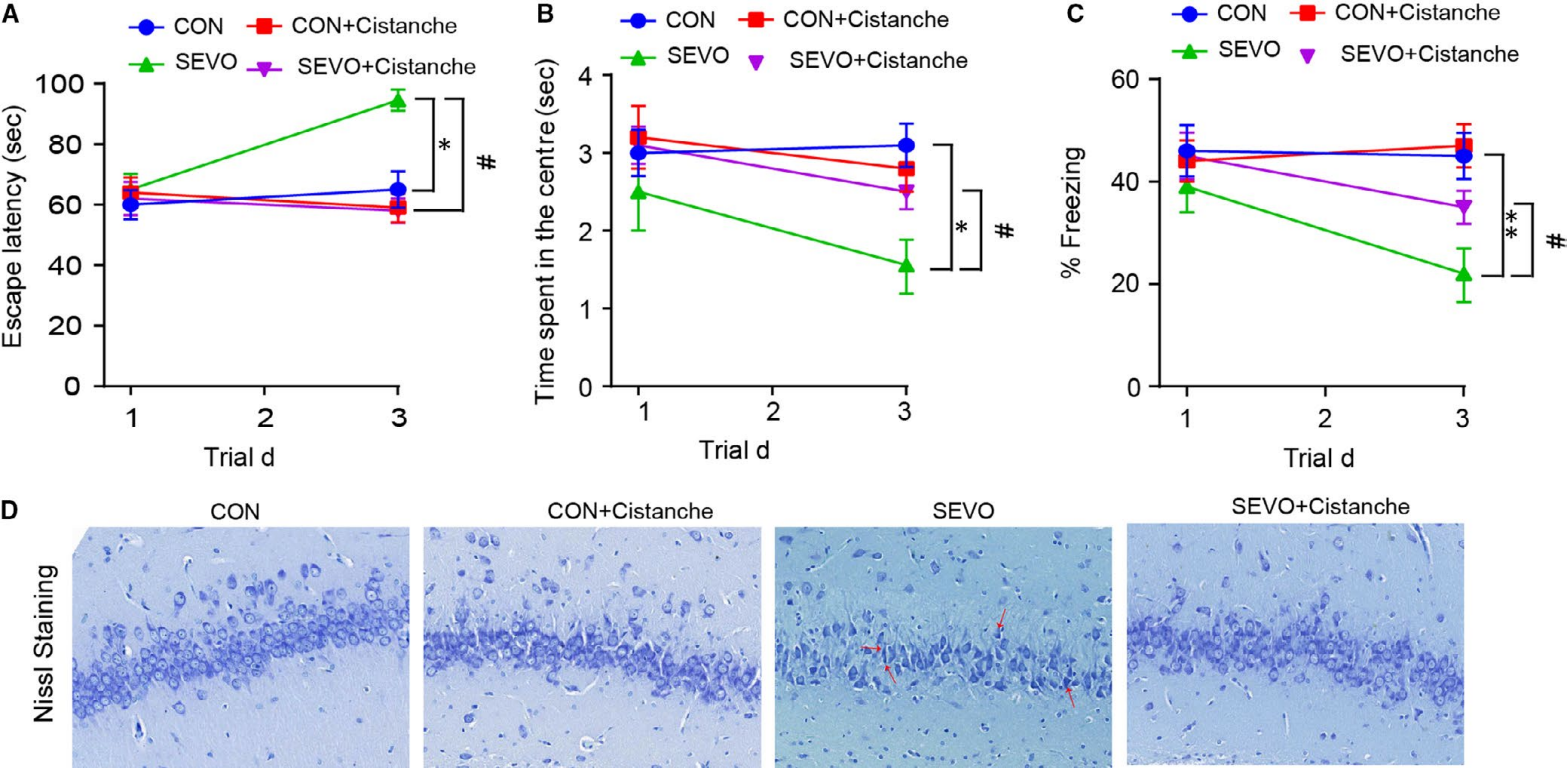
Cistanche ameliorates the cognitive decline. A, The curves show the latency to locate the platform for day 1 and day 3. B, Time spent in the centre for animals treated with cistanche (50 mg/kg) and saline in the open‐field test (OFT). C, Percentage of time the rats froze during the 5‐min context test on day 1 and day 3 of the FC test. D, Nissl staining of rats' hippocampal tissues (CA1 region) on day 3. Data are expressed as mean ± SD (n = 8 per group), *P < .05, **P < .01 vs CON group; ^#^ *P* < .05 vs SEVO group

To further investigate the role of cistanche on the cognitive function, rats were subjected 50 mg/kg of cistanche orally for three consecutive days prior to anaesthesia. In the cistanche‐treated group (group SEVO + cistanche), the escape latency was significantly shortened, the time of spent in the centre was longer, and the per cent of freezing was increased at day 3 after anaesthesia when compared to group SEVO (Figure 5A‐C). These results indicated that cistanche could at least partially rescue cognition impairments. We further evaluated the extent of neuronal death in the hippocampus of rats in four groups by Nissl staining. Images presented that cells had no obvious abnormality in group control and group control + cistanche. However, the number of survival neurons in the CA1 regions was significantly reduced in the SEVO‐induced rats compared with that in the control group on day 3. Treatment with cistanche reversed this effect of SEVO and significantly promotes neuronal survival compared with SEVO‐induced alone (Figure 5D).

### 3.6 Cistanches Herba extract treatment reduced sevoflurane‐induced pro‐inflammatory cytokine production in a PPAR‐γ‐sensitive manner

**Figure 6:**
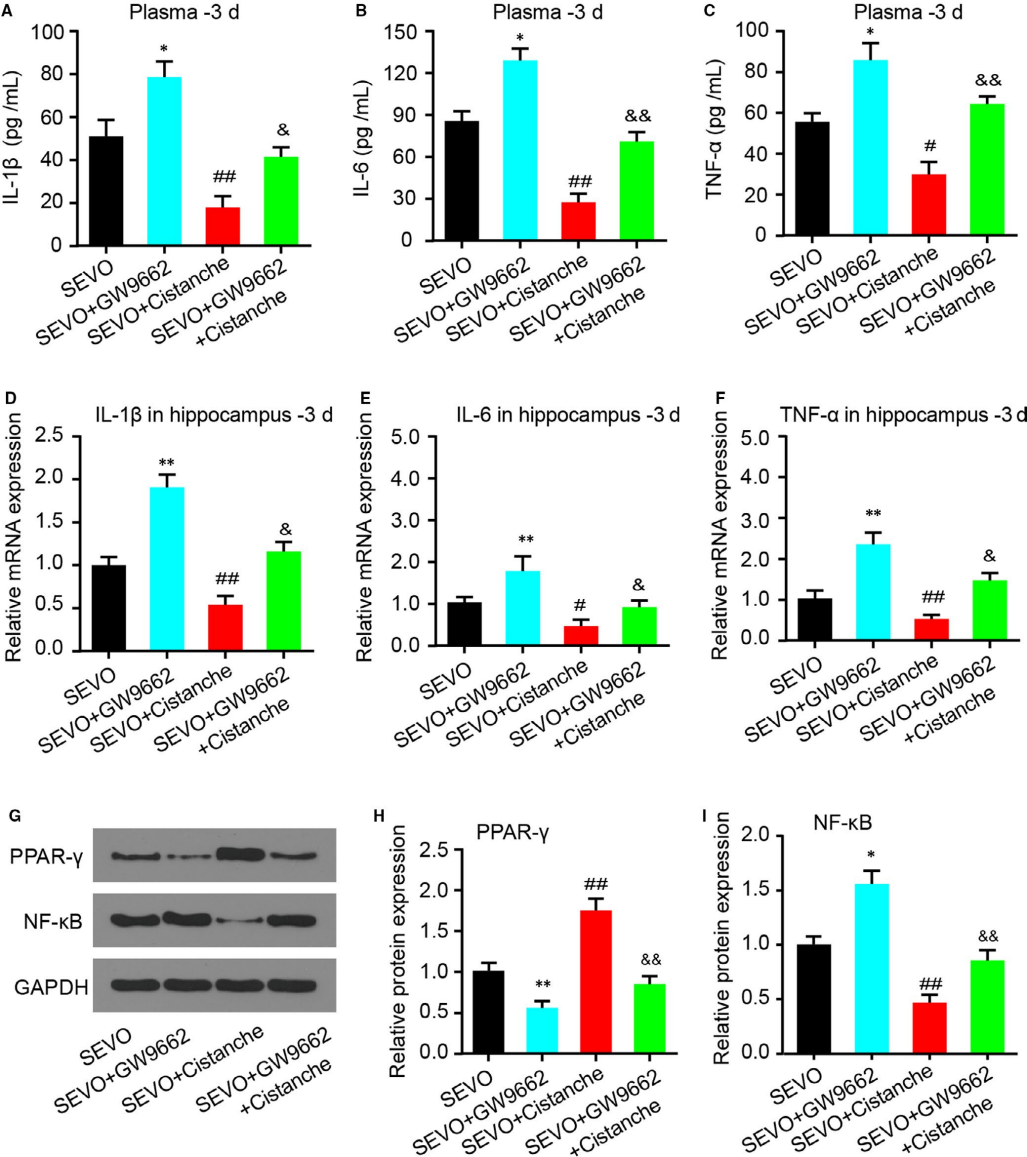
Cistanche inhibits sevoflurane‐induced neuroinflammation in a PPAR‐γ‐sensitive manner. A‐C, The concentration of pro‐inflammatory factors IL‐1β and IL‐6 in rat plasma samples on day 3 was measured by ELISA. D‐F, Relative mRNA levels of IL‐1β, IL‐6 and TNF‐α in the hippocampus on day 3 after treatment were evaluated by qRT‐PCR. G, The levels of NF‐κB and PPAR‐γ in the hippocampus were detected by Western blotting using specific antibodies. (H and I) The expression of NF‐κB and PPAR‐γ was quantified and normalized to GAPDH levels. Each value was then expressed relative to the SEVO, which was set to 1. *P < .05 and **P < .01 vs SEVO group; ^#^ *P* < .05, ^##^ *P* < .01 vs SEVO group; ^&^ *P* < .05 and ^&&^ *P* < .01 vs SEVO + cistanche group

We next investigated whether the effect of cistanche on cytokine production was PPAR‐γ‐sensitive. Treatment with GW9662 (group SEVO + GW9662) increased the levels of IL‐1β, IL‐6 and TNF‐α compared to the SEVO group. However, treatment with cistanche diminished SEVO‐induced pro‐inflammatory cytokine production. Of note, the effects facilitated by cistanche were reverted by GW9662, as seen by a reduction in these same cytokines (Figure 6A‐F). As aforementioned, cistanche treatment promoted the expression of PPAR‐γ and suppressed NF‐κB expression, we therefore detected the expression of PPAR‐γ and NF‐κB to determine the role of PPAR‐γ signalling in the pharmaceutical effect of cistanche in anaesthetic aged rats. SEVO + GW9662 decreased PPAR‐γ level and increased the expression of NF‐κB in the hippocampus strikingly, which was attenuated by administration of cistanche (Figure 6G‐I).

### 3.7 Cistanches Herba extract treatment inhibited sevoflurane‐induced oxidative stress via PPAR‐γ

**Figure 7:**
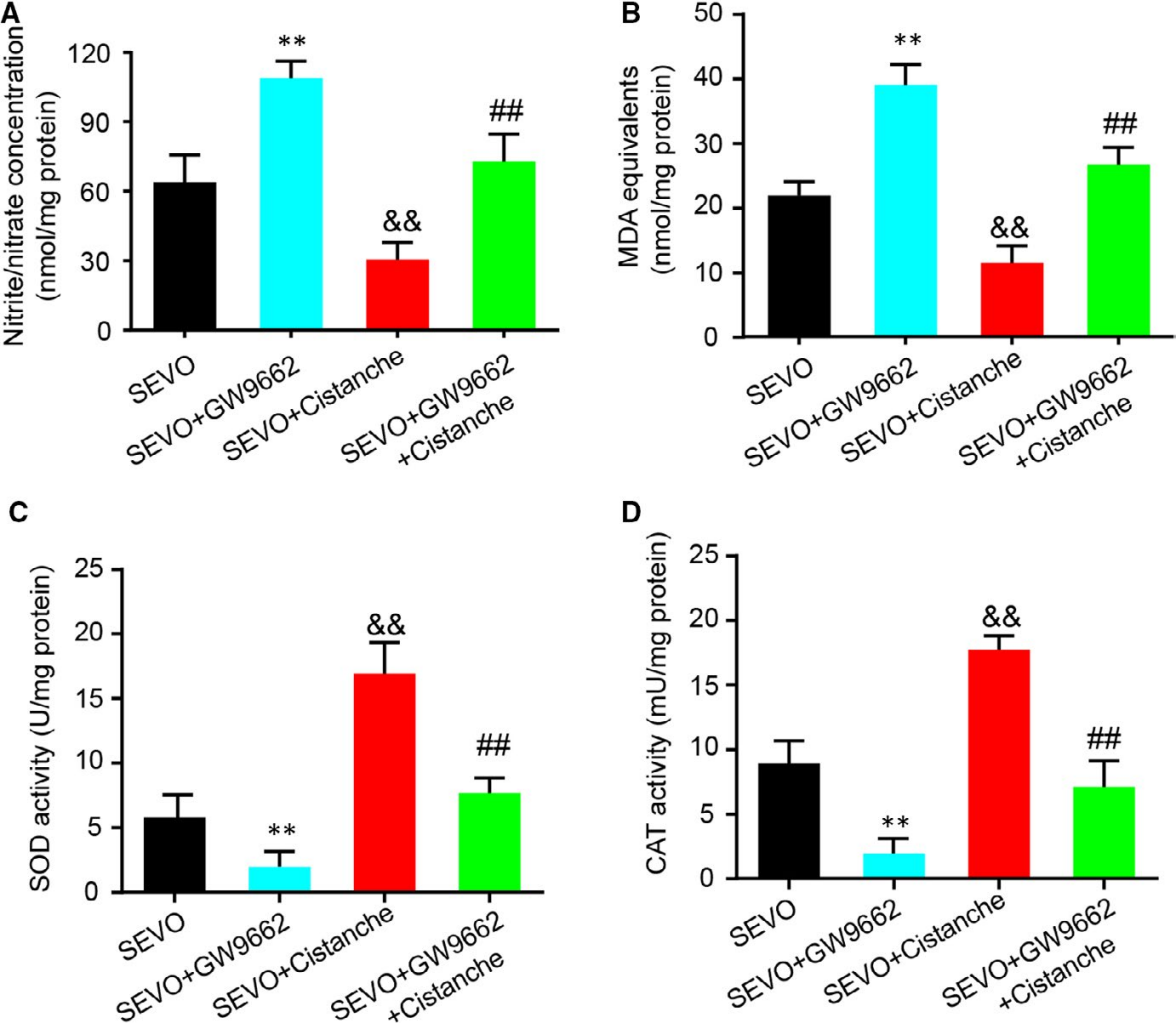
Cistanche inhibits the oxidative stress induced by sevoflurane in a PPAR‐γ‐dependent manner. Three days after sevoflurane induction, hippocampal tissues were collected for the determination of nitrite/nitrate concentration (A) and lipid peroxidation (B), SOD levels (C) and CAT levels (D). Results are mean ± SD, n = 8 rat per group in each experiment, **P < .01 vs SEVO group; ^##^ *P* < .01 vs SEVO group; ^&&^ *P* < .01 vs SEVO + cistanche group

It had been reported that sevoflurane exaggerates cognitive dysfunction in a chronic intermittent hypoxia‐induced rat model through inhibition of PPAR‐γ expression in the hippocampus.^1^ We next wondered whether the effect of cistanche occurred in a PPAR‐γ‐dependent manner. For this purpose, GW9662, a selective and irreversible antagonist of PPAR‐γ, was used for further study. Treatment with GW9662 at 30 ng promoted the oxidative stress, as demonstrated by the increased levels of nitrite/nitrate and MDA. However, the anti‐oxidative markers SOD and CAT in hippocampus on day 3 were increased compared to SEVO‐operated rats. Treatment with cistanche reduced SEVO‐induced overproduction of oxidative stress markers, and facilitated the SOD and CAT activity (Figure 7A‐D). Furthermore, these effects produced by cistanche were reverted by GW9662, as observed by an increase in nitrite/nitrate (Figure 7A) and MDA (Figure 7B) production. In addition, the effects of cistanche on SOD and CAT activity were also reverted by GW9662 (Figure 7C, D), indicating the participation of PPAR‐γ.

### 3.8 Cistanches Herba extract treatment inhibited microglia activation and neurocyte apoptosis and ameliorated the cognitive decline in a PPAR‐γ‐sensitive manner

**Figure 8:**
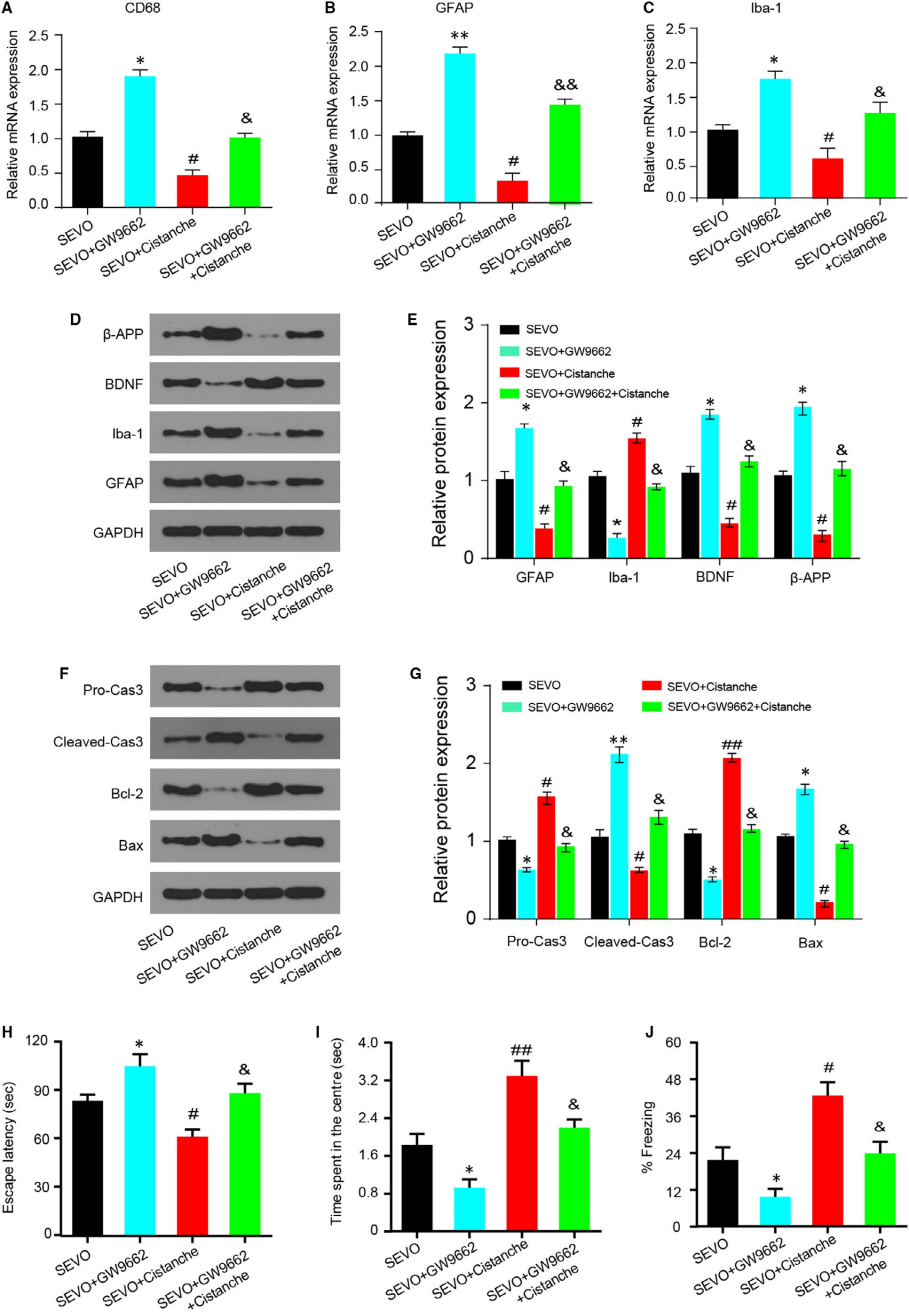
Cistanche inhibits microglia activation and neurocyte apoptosis and ameliorates the cognitive decline in a PPAR‐γ‐sensitive manner. A‐C, The mRNA levels of glial markers CD68, GFAP and Iba‐1 were measured by qRT‐PCR. D, E, The protein β‐APP, BDNF, Iba‐1 and GFAP levels on day 3 after cistanche administration were examined by Western blot. F, The expression of pro‐caspase 3, cleaved‐caspase 3, Bcl‐2 and Bax was detected by Western blotting using specific antibodies. G, Relative expression of pro‐caspase 3, cleaved‐caspase 3, Bcl‐2 and Bax was quantified and normalized to GAPDH levels. The escape latency to land on the platform (H) and time spent during the original platform quadrant (I) was measured. J, Performance during fear conditioning tests on day 3 after treatment. Results are mean ± SD, n = 8 rat per group in each experiment, *P < .05 and **P < .01 vs SEVO group; ^#^ *P* < .05 and ^##^ *P* < .01 vs SEVO group; ^&^ *P* < .05 and ^&&^ *P* < .05 vs SEVO + cistanche group. ^&^ *P* < .05 vs SEVO + cistanche group

The next step was to explore whether the effects of cistanche on microglia activation and neurocyte apoptosis were PPAR‐γ‐sensitive. Treatment with GW9662 increased the mRNA levels of CD68 (Figure 8A), GFAP (Figure 8B) and Iba‐1 (Figure 8C). Notably, these effects produced by GW9662 were effectively reverted by cistanche, as observed by a decrease in these same markers (Figure 8A‐C). Moreover, the protein expression of GFAP and Iba‐1 produced by GW9662 was also reverted by cistanche, indicating that cistanche inhibits microglia activation, and at least partially depends on PPAR‐γ. Additionally, the effect of cistanche on β‐APP and BDNF protein expression was inhibited by GW9662 (Figure 8D, E). To investigate whether cistanche could inhibit neurocyte apoptosis in a PPAR‐γ‐sensitive manner, the apoptosis‐related proteins were detected. As shown in Figure 8F, G, cistanche at 50 mg/kg reduced the expression of pro‐apoptosis protein (cleaved‐caspase 3 and Bax). However, these effects were largely rescued by GW9662.

Since the oxidative stress and inflammation levels were closely related to cognitive function in post‐anaesthesia rats, we further determined whether the effect of cistanche on cognitive dysfunction was PPAR‐γ‐dependent. As shown in Figure 8H‐J, compared with rats in the SEVO group, the escape latency in group SEVO + GW9662 was markedly prolonged, while the time spent in the centre was shortened, and the per cent of freezing time was decreased at day 3. However, compared with the rats treated with GW9662, co‐treated with GW9662 and cistanche, a significantly increased cognition was observed, with the shortened escape latency (Figure 8H), the prolonged time of spending in the centre (Figure 8I) and the increased percent of freezing time at day 3 (Figure 8J). These results demonstrated that the protective effects of cistanche in sevoflurane‐induced cognitive dysfunction were compromised when PPAR‐γ was inhibited, suggesting that cistanche at least partially exerts its protective effects by the activation of PPAR‐γ.

## 4 DISCUSSION

In this study, the findings demonstrated that a 2.6% concentration of sevoflurane exposure during 4 hours had notable negative effects on the learning and memory abilities of aged rats. Remarkably, a single‐dose cistanche (50 mg/kg) could exert anti‐inflammatory, antioxidant, anti‐apoptosis, anti‐activation of microglia and neuroprotection effects on the sevoflurane‐induced aged rats by activating PPAR‐γ signalling.

Sevoflurane, a volatile anaesthetic, is most commonly used in patients of all ages in modern anaesthesiology. However, several investigates have reported that sevoflurane may lead to cognitive dysfunction, and these negative effects may depend on the concentration of sevoflurane, the duration and number of anaesthetic exposures. For instance, Xu et al^10^ have described that short‐term exposure to sevoflurane‐impaired working memory in aged rats. In their study, memory impairment of aged rats reduced at day 1 after exposure to 1 minimum alveolar concentration (MAC) sevoflurane and at days 1, 3 and 7 after exposure to 1.5 MAC sevoflurane, suggesting that the extent of cognitive function impairment was related to its concentration. Despite elderly adults are more sensitive to the cognitive impairment effects of sevoflurane due to the reduced physical function to compete the stress,^24^ cognitive dysfunction also appears in adults. In the present study, we demonstrated that 24‐ but not 3‐month‐old rats have cognitive impairments on day 3 after the induction of anaesthesia with 2.6% sevoflurane in 100% oxygen during 4 hours, indicating that the negative effects of the sevoflurane on the cognitive function are age‐related under this condition.

Numerous studies have reported that surgery and anaesthesia have been associated with a transient or permanent damage in cognitive function, whereas the aetiology of the disorders is largely unknown.^25^ Neuroinflammatory and oxidative stresses are the key players in cognitive impairment after surgery. On one side, neuroinflammation is the primary source of reactive oxygen species (ROS), free radicals, and reactive nitrogen species in the activated central nervous system.^26^ In turn, the excess ROS produced can damage biomolecules, change cellular functions and promote inflammation consequently.^27^ Neuroinflammation has been suggested to be a cornerstone of many neurodegenerative and cognitive diseases, including POCD.^2^, ^28^ Gong has described that sevoflurane exposure impaired spatial memory in aged rats by increasing inflammation.^29^ Sevoflurane inhalation enhances the release of cytokines (IL‐1β, IL‐6 and TNF‐α) and the activation of NF‐κB signalling in the hippocampus of adult rats.^30^ On the other hand, the elevated antioxidant defence and apoptosis status were also observed in blood samples of sevoflurane‐administered children.^31^ In contrast, studies have shown that a widely used agent (dexamethasone) in the perioperative course can inhibit inflammatory events, prevent the production of IL‐6 and make alterations in learning and memory processes.^8^, ^32^ Ye and his colleague reported that honokiol could alleviate surgery/anaesthesia‐induced cognitive dysfunction in mice through regulation of neuroinflammatory and oxidative stress in hippocampus.^23^

Cistanches is a commonly used Traditional Chinese Medicine (TCM). Although several pharmacological studies have shown that cistanches exhibited anti‐inflammatory, immunomodulatory, anti‐fatigue, anti‐oxidative anti‐tumour hepatoprotection and neuroprotective effects,^13^ whether cistanches manipulates the effect of sevoflurane‐induced inflammatory and oxidative stress remains unknown. Here, we demonstrated that Cistanches Herba extract reduces the expression of IL‐1β, IL‐6, TNF‐α and NF‐κB in the hippocampus‐induced rats. Administration of Cistanches Herba extract directly reduces the nitrite/nitrate and MDA concentration and increases the SOD and CAT activity. These data suggested that treatment with Cistanches Herba extract significantly ameliorated the neuroinflammation and oxidative stress induced by sevoflurane exposure, indicating an intervention strategy for sevoflurane anaesthesia.

In addition, neuroinflammation is thought to be regulated by microglia, the resident immune cells in the brain.^33^ Upon stimulation, microglia become gradually activated and produce numerous pro‐inflammatory cytokines that may cause neurodegeneration.^34^ What's more, BDNF has a significant role in promoting the synthesis and consolidation of new memories,^35^ and neuroinflammation and oxidative stress can inhibit the brain‐derived neurotrophic factor (BDNF) expression.^36^ Here, our data demonstrated that administration of Cistanches Herba extract inhibited microglia activation from sevoflurane induction as indicated by a reduced expression of GFAP and Iba‐1, and increased BDNF levels in the hippocampus, suggesting that inhibiting the microglia activation appears to be a mechanism to reduce inflammation in the hippocampus.

Increased oxidative stress and inflammation to several tissues/organs lead to cell death and long‐term injury. Studies showed that sevoflurane anaesthesia could reduce the rate of neurogenesis and decrease neuronal survival in the hippocampus, causing neurotoxicity and cognitive impairment in aged rats.^37^, ^38^ Moreover, an in vitro study demonstrated that inhalation of sevoflurane could increase β‐AP levels and induce caspase activation and apoptosis, which can ultimately facilitate the progression of Alzheimer's disease.^39^ Additionally, it is also revealed that exposure sevoflurane in aged rats led to learning and memory deficits through endoplasmic reticulum stress (ERS)‐induced apoptosis of the neurons.^40^ We found that treatment with Cistanches Herba extract down‐regulated the expression of apoptosis‐related proteins, namely cleaved‐caspase 3 and Bax. Meanwhile, Cistanches Herba extract promoted the expression of anti‐apoptotic protein Bcl‐2 in the hippocampal region of sevoflurane‐induced aged rats. Thus, our findings reveal a function for Cistanches Herba extract as a neuroprotective agent for the survival of neurone.

Remarkably, PPAR‐γ (a ligand‐inducible transcription factor of the nuclear hormone receptor superfamily) is known to play an important role in the inflammatory response.^41^ The ability of PPAR‐γ to exert anti‐inflammatory effects has been extensively studied,^42^, ^43^, ^44^ Moreover, activation of PPAR‐γ exerts anti‐inflammatory effects via inhibiting the transcription factors, such as activator protein‐1 and nuclear factor‐kB.^41^ In addition, inhibiting PPAR‐γ by its antagonists exhibits protective effects in an ischaemia/reperfusion injury rat model by preventing oxidative stress and excessive inflammatory response.^45^ Chen et al demonstrated that thymoquinone inhibits spinal cord injury via preventing inflammatory response, apoptosis and oxidative stress through PPAR‐γ and PI3K/Akt pathways.^46^ What’ more, PPAR‐γ is expressed in various cell types including astrocytes, microglia and neurons in the brain, and PPAR‐γ activation in microglia leads to reduce the production of pro‐inflammatory cytokines by these cells.^47^ Notably, sevoflurane exaggerates cognitive dysfunction by increasing microglia‐mediated neuroinflammation through inhibition of PPAR‐γ in the hippocampus in a chronic intermittent hypoxia rat model.^1^ Furthermore, Xiang *et al* found that down‐regulation of miR‐27a‐3p could alleviate sevoflurane‐induced neurotoxicity and improve learning and memory abilities by mediating the PPAR‐γ signalling pathway.^48^ Cistanches was previously demonstrated to show anti‐inflammatory, antioxidant, anti‐activation of microglia and neuroprotection effects, but a comprehensive investigation on the mechanism of it is still lacking. Besides, previous studies revealed that cistanches exerted anti‐inflammatory function by regulating the NF‐κB and TGF‐β signalling,^13^ but whether cistanches manipulates PPAR‐γ signalling to regulate inflammatory and oxidative stress and protect cognitive function remains unknown. In this study, we found that Cistanches Herba extract prevented the inflammatory, oxidative stress, glia activation, apoptosis and cognitive dysfunction at least partially via PPAR‐γ signalling pathway. In support of its functional essentiality, a PPAR‐γ antagonist GW9662 was used combined with Cistanches Herba extract in vivo. Results showed that inhibition of PPAR‐γ markedly alleviated the effectivity of Cistanches Herba extract in treating rats in vivo, indicating the potential of the PPAR‐γ‐activated therapeutic approach (Figure S2).

Although Cistanches Herba extract had revealed to relieve cognitive impairment induced by sevoflurane anaesthesia in aged male rats in vivo by activating PPAR‐γ signalling, the relevance between Cistanches Herba extract and other signalling pathways needs more validation. Moreover, the effects of Cistanches Herba extract on age‐matched female rats or the younger males still deserve further investigation.

## 5 CONCLUSIONS

Our studies demonstrated that Cistanches Herba extract, through activating the PPAR‐γ signalling, alleviated the sevoflurane anaesthesia‐induced cognitive impairment in aged rats. Our results provided a potential strategy for preventing the POCD induced by sevoflurane anaesthesia clinically.

## CONFLICT OF INTEREST

The authors confirm that there are no conflicts of interest.

## AUTHORS' CONTRIBUTIONS

Substantial contribution to the conception and design of the work: SP, PYL, PRL, JW; Analysis and interpretation of the data: SP, HZY, WHL, CLL, YXZ; Drafting the manuscript: SP, PYL, HY, YXZ; Revising the work critically for important intellectual content: YXZ; Collection of grants: YXZ; Final approval of the work: all authors.

## ETHICS APPROVAL AND CONSENT TO PARTICIPATE

All procedures performed in studies involving animals were in accordance with the ethical standards of Seventh People's Hospital of Shanghai University of TCM.

## CONSENT FOR PUBLICATION

This manuscript has been approved by all authors for publication.

## Data Availability

The data sets used and analysed during the current study are available from the corresponding author on reasonable request.
