# Supplementary figures and images for: Cistanches alleviates sevoflurane‐induced cognitive dysfunction by regulating PPAR‐γ‐dependent antioxidant and anti‐inflammatory in rats
Source: J Cell Mol Med. 2019 Dec 4;24(2):1345–59. doi: 10.1111/jcmm.14807 (PMC6991648; doi:10.1111/jcmm.14807)

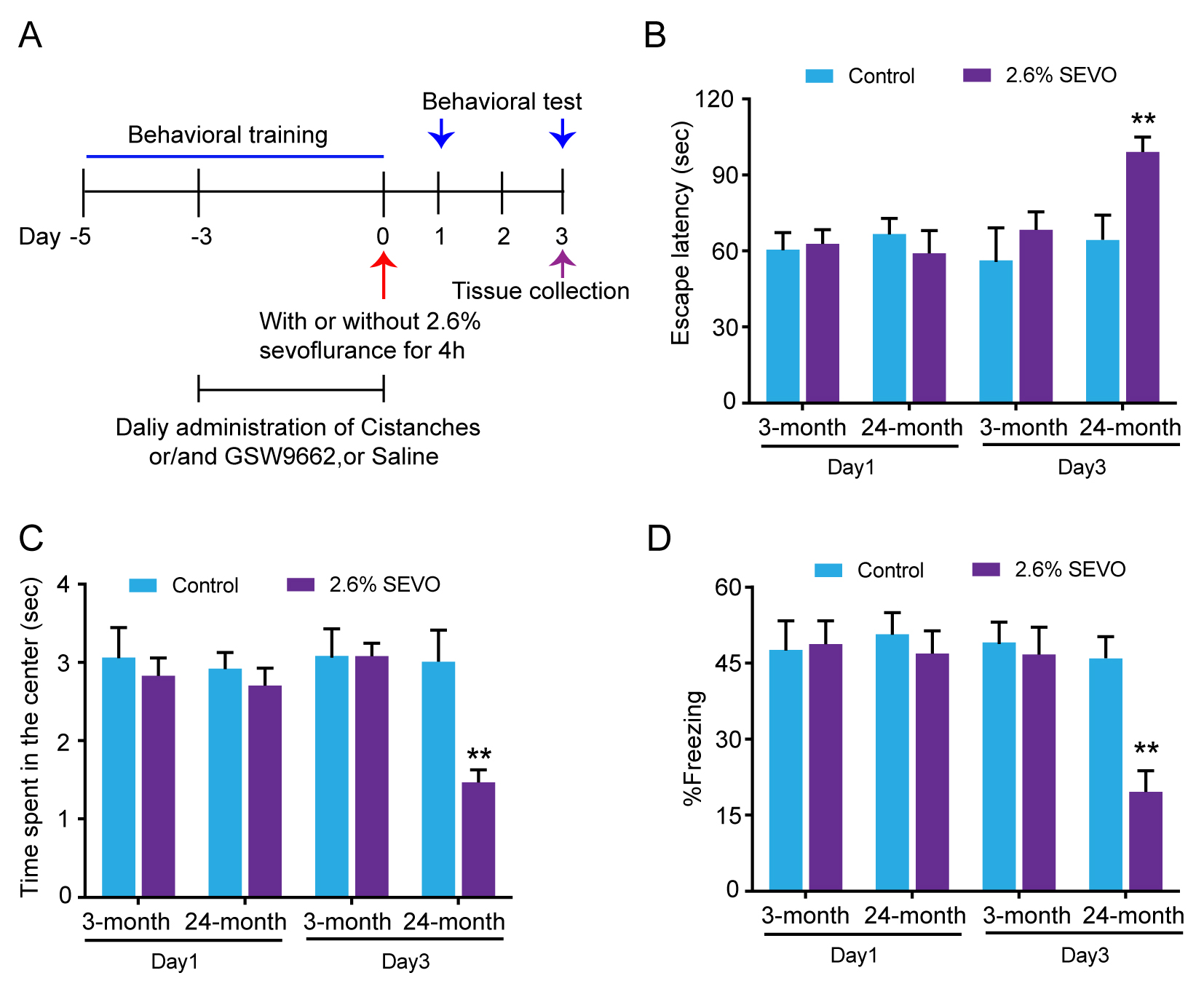

Supplement: Supplementary file 1 [file JCMM-24-1345-s001.jpg]

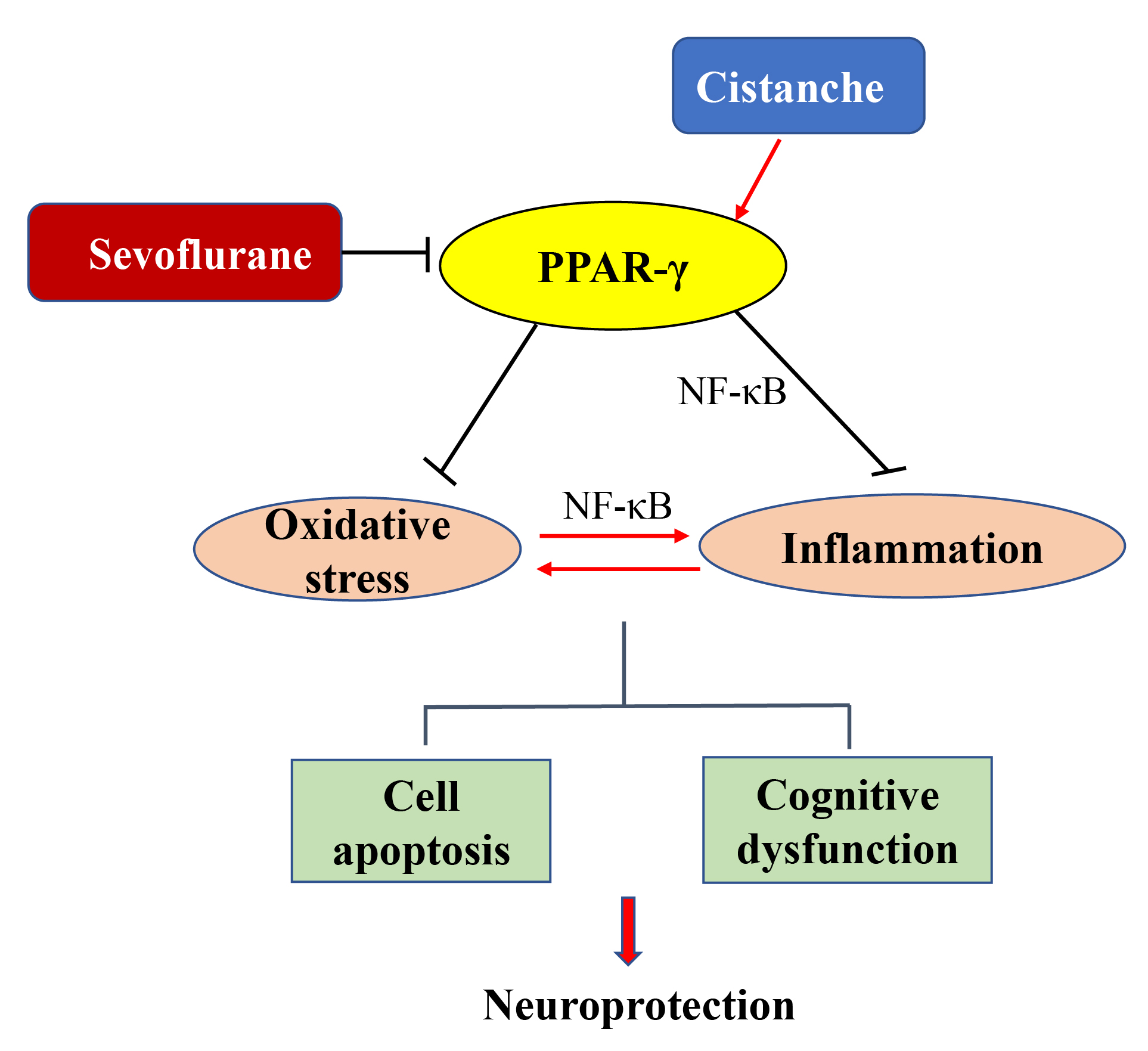

Supplement: Supplementary file 2 [file JCMM-24-1345-s002.jpg]
