# Supplementary material for: Cistanches alleviates sevoflurane‐induced cognitive dysfunction by regulating PPAR‐γ‐dependent antioxidant and anti‐inflammatory in rats
Source: J Cell Mol Med. 2019 Dec 4;24(2):1345–59. doi: 10.1111/jcmm.14807 (PMC6991648; doi:10.1111/jcmm.14807)
Supplement: Supplementary file 3 [file JCMM-24-1345-s003.docx]

**Table S1. The primers of qRT-PCR**

| Gene | Accession in Gene bank | Primer sequences | Amplicon size (bp) |
| --- | --- | --- | --- |
| IL-1β | NM_031512.2 | Forward: 5’-GACCTGTTCTTTGAGGCTGACA-3’  Reverse: 5’-CTCATCTGGACAGCCCAAGTC-3’ | 78 |
|  |  |  |  |
| IL-6 | NM_012589.2 | Forward: 5’-TAGTCCTTCCTACCCCAACTTCC-3’  Reverse: 5’-TTGGTCCTTAGCCACTCCTTC-3’ | 76 |
|  |  |  |  |
| TNF-α | NM_012675.3 | Forward: 5’-GACCCTCACACTCAGATCATCTTCT-3’  Reverse: 5’-TGCTACGACGTGGGCTACG-3’ | 64 |
| CD68 | NM_001031638.1 | Forward: 5’-ACCCGGAGACGACAATCAAC-3’ | 70 |
|  |  | Reverse: 5’-CTTGGTGGCCTACAGAGTGG-3’ |  |
|  |  |  |  |
| GFAP | NM_017009.2 | Forward: 5’-CTGAAAGTGTCCCCTCAGTT-3’ | 113 |
|  |  | Reverse: 5’-ACAGTACTGCTCTGAAGGTTAG-3’ |  |
|  |  |  |  |
| Iba-1 | NM_017196.3 | Forward: 5’-CCAGCGTCTGAGGAGCTATG-3’ | 173 |
|  |  | Reverse: 5’-CGTCTTGAAGGCCTCCAGTT-3’ |  |
|  |  |  |  |
| GAPDH | NM_017008.4 | Forward: 5’-CATGGCCTTCCGTGTTCCTA-3’  Reverse: 5’-GCGGCACGTCAGATCCA-3’ | 55 |
